# Antegrade selective cerebral perfusion in patients with "bovine aortic arch": is it easier?

**DOI:** 10.1186/1749-8090-3-60

**Published:** 2008-11-04

**Authors:** Federico Bizzarri, Consalvo Mattia, Matteo Di Nardo, Emanuele Di Marzio, Massimo Ricci, Flaminia Coluzzi, Giacomo Frati, Paolo Pagliaro, Luigi Muzzi, Vincenzo Petrozza

**Affiliations:** 1 Cardiac Surgery Unit-Polo Pontino, Heart and Great Vessels Department, University of Roma "Sapienza", Via F. Faggiana 34, Latina-Italy; 2 Anaesthesiology and Intensive Care Unit, Rome University "Sapienza"-Polo Pontino, Via F. Faggiana 34, Latina-Italy; 3 Pathology Unit, Rome University "Sapienza", Via F. Faggiana 34, Latina-Italy

## 

The term "bovine aortic arch" is widely used to describe a common anatomic variant of the human aortic arch branching, regarding the common origin of the brachiocephalic and left common carotid artery from the aorta. This anatomy pattern is not generally found in the cattles and the term "bovine aortic arch" is a common misnomer in the medical literature. Nevertheless we use this term all the time we deal with this kind of arch anatomy. True incidence is not really known and it's not clear if an incidental correlation exists between this arch anomaly and bicuspid aortic valve with ascending aorta aneurysms or if an anatomic predisposition identifies a sort of syndrome involving the left ventricular outflow tract.

We report the medical records of 3 consecutive patients, with this anomaly, undergone surgical treatment of bicuspid aortic valve pathology and ascending aorta aneurysm under profound hypothermia and selective cerebral perfusion (SCP) through the right axillary artery.

Bicuspid aortic valve affects about 2% of the population and is associated with connective tissue disorder that predisposes to thoracic aorta dilatation (Fig 1 and 2) and increased risk of aortic dissection and rupture [1]. Therefore, the surgery of a pathologic bicuspid aortic valve implies the treatment of ascending aorta and/or arch.

**Figure 1:**
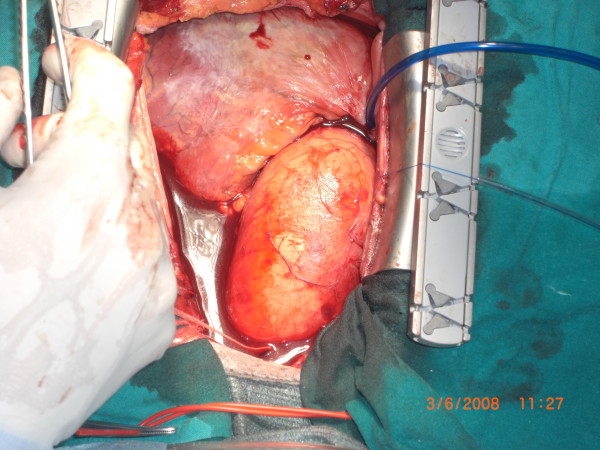
intraoperative picture of dilated aorta.

**Figure 2:**
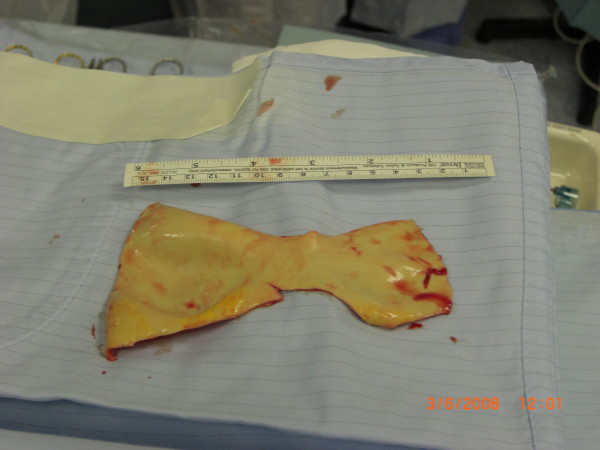
macroscopic specimen of the aorta's intima.

We describe the outcomes of 3 consecutive patients seen with these anomalies treated with the same surgical technique at Cardiac Surgery Unit of Polo Pontino between March and April 2008.

## Case reports

### Patient 1

This 62-year old man presented with signs and symptoms of acute pulmonary congestion. Echo evaluation revealed a bicuspid aortic valve with 4+ regurgitation, 45% ejection fraction and increased diameters of left ventricle. 64 slices CT scan evaluation showed a 7.5 cm dilatation of ascending aorta involving the proximal portion of the arch and "bovine" configuration of the arch. Coronary angiography revealed a 80% stenosis of descending anterior artery.

### Patient 2

This 69-year-old man presented with exertion dyspnea. Echo evaluation revealed a bicuspid aortic valve with 3–4+ regurgitation, 35% ejection fraction. 64 slices CT scan evaluation showed a 6.5 cm dilatation of ascending aorta involving the proximal portion of the arch and "bovine" configuration of the arch. Coronary angiography showed a normal coronary tree.

### Patient 3

This 59-year-old man presented with thoracic pain and exertion dyspnea. Echo evaluation revealed, as the other patients, a bicuspid aortic valve with 4+ regurgitation and 40% ejection fraction. 64 slices CT scan evaluation showed a 8.0 cm dilatation of ascending aorta extending to the proximal portion of the arch and "bovine " configuration of the arch. Coronary angiography revealed a two vessel disease, 85% stenosis of descending anterior artery and 75% stenosis of the right coronary artery.

### Surgical Technique

Operations were performed through a median sternotomy after isolation of the right axillary artery at the deltoideus-pectoralis groove. After systemic heparinization, a cardiopulmonary bypass (CPB) was instituted with an arterial cannula introduced into the right axillary artery and with a venous single two-stage cannula introduced into the right atrium. Myocardial protection was achieved with a cold cristalloid cardioplegia (Bretschneider solution) through the coronary sinus and through the coronary ostia. Cerebral monitoring was achieved by means of noninvasive measure of brain oxygenation regional saturation of oxygen (rSO_2_, INVOS Cerebral Oximeter System, Somanetics Corporation, Troy, Michigan, USA). The patients were cooled down to a nasopharyngeal temperature of 25°C. Arterial blood pH was managed according to the α-stat method.

After the arrest, antegrade selective cerebral protection (ASCP) was performed according to Kazui et al. protocol [2] by clamping the origin of the "bovine trunk". Cerebral perfusion was initiated at a flow rate of 10 ml/Kg.

An open distal anastomosis was first performed, then ASCP was stopped and the systemic circulation restored and the patient rewarmed. Ascending aorta and valve were replaced by means a valved tube with coronary reimplantation (modified Bentall procedure) and coronary artery grafting, at least, in patient 1 and 3. In all the patient the average of ASCP time was 20 ± 3.3 minutes.

## Comment

Antegrade selective cerebral perfusion is a safe technique for thoracic aorta surgery allowing "difficult" aortic repairs to be performed with good results in terms of hospital mortality and neurologic outcomes [3]. The modern techniques to achieve cerebral protection include deep hypotermia, right axillary artery cannulation and perfusion of carotid arteries by means of cannulae inserted into a single artery or in both of them. In special situation carotid artery cannulation is a fast, safe and efficient method to achieve cerebral protection, but it provides an unilateral perfusion of carotid arteries [4]. Cannulation of the femoral artery [5] remains a standard procedure in acute type A aortic dissection and other aortic diseases, but it should be avoided in the dissections involving the iliac or femoral arteries or in severe atherosclerotic disease of aorta.

Sometimes a "special" human arch anatomy simplifies the methods to achieve cerebral perfusion allowing bilateral carotid perfusion without insertion of cannulae leaving totally free the surgical field to perform an open distal anstomosis in a safe and not time-consuming way.

The most common aortic arch branching pattern in humans consists of 3 great vessels arising from the arch of the aorta. The second most common variant occurs when the left common carotid artery has a common origin with the innominate artery and this variant is most often termed as a "bovine aortic arch". A similar but less common variant occurs when the left common carotid artery originates directly from the innominate artery rather than as a common trunk [6].

In our patients cerebral protection has been achieved taking advantage of this kind of anatomy that offers the opportunity to simplify a complex procedure providing a bilateral cerebral perfusion without cannulae or catheters into the carotid arteries.

## Authors' contributions

FB, GF, MR and LM were the surgeons. CM, MDN, EDM, FC and PP were the anesthesists involved in theatre and in the postoperative care. VP was the pathologist. All authors have read and approved the final manuscript.

## Consent

Written informed consent was obtained from the patients for publication of this case report and the accompanying image. A copy of the written consent is available for review by the Editor-in-Chief of this journal.
